# A case of synchronous association of a jejunal stromal tumor and a colic adenocarcinoma

**DOI:** 10.11604/pamj.2019.34.197.19373

**Published:** 2019-12-16

**Authors:** Refeno Valéry, Hasiniatsy Nomeharisoa Rodrigue Emile, Ramahandrisoa Andriatsihoarana Voahary Nasandratriniavo, Ramanampamonjy Rado Manitrala, Samison Luc Hervé, Rafaramino Florine

**Affiliations:** 1 Department of Oncology, Professor Zafisaona Gabriel Teaching Hospital, Mahajanga, Faculty of Medicine of Mahajanga, Mahajanga , Madagascar; 2 Department of Oncology and Palliative Care, Military Hospital Antananarivo, Faculty of Medicine of Antananarivo, Antananarivo, Madagascar; 3 Department of Oncology, Joseph Ravoahangy Andrianavalona Teaching Hospital Antananarivo, Faculty of Medicine of Antananarivo Antananarivo, Madagascar; 4 Department of Gastro-Enterolgy, Joseph Raseta Befelatanana Teaching Hospital Antananarivo, Faculty of Medicine of Antananarivo, Antananarivo, Madagascar; 5 Department of Visceral surgery, Joseph Ravoahangy Andrianavalona Teaching hospital Antananarivo, Faculty of Medicine of Antananarivo, Antananarivo, Madagascar; 6 Faculty of Medicine of Antananarivo, Antananarivo, Madagascar

**Keywords:** Adenocarcinoma, association, stromal tumors, surgery, synchronous

## Abstract

Gastrointestinal stromal tumors are the most common mesenchymal tumors of the gastrointestinal tract. They are generally considered as solitary tumors. Here we report the case of a 66-year-old man with familial history of digestive cancers, admitted after a 2-months history of gastrointestinal bleeding. Colonoscopy showed a circumferential ulcerated tumor of the sigmoid which was a well differentiated adenocarcinoma on biopsy. Computerized tomography showed only the lesion of the sigmoid. The patient underwent a left colectomy followed by immediate end-to-end anastomosis. Per-operatively, jejunal mass was discovered and a jejunal segmentectomy was performed. Pathologic examination revealed a colic adenocarcinoma pT2N0M0 associated with a low risk stromal tumor of the jejunum CD117+ and DOG-1+. No adjuvant therapy was given. After 64 months of follow-up, the patient showed no evidence of recurrence. Clinicians should be aware of the coexistence of malignancies and carry careful investigations for accurate diagnosis and better patients' prognosis.

## Introduction

Gastrointestinal Stromal Tumors (GIST) are mesenchymal tumors developed from the interstitial cells of Cajal (ICC). They express C-KIT (CD117) in 95% of cases and may also express other markers such as DOG-1, S100 protein, CD34, BCL-2 and desmine which allow differentiate them from other mesenchymal tumors [1]. They represent about one percent of the gastro-intestinal tumors [2, 3]. The preferred localization of this tumor are the stomach, followed by small intestine, colon-rectum and esophagus [4]. GIST are generally considered as solitary tumors but they can be associated with other gastro-intestinal tumor [3, 5]. The association of a jejunal stromal tumor with a colic adenocarcinoma is relatively uncommon and no previous case has been reported in Madagascar. We report a case of jejunal stromal tumor discovered incidentally during operation for colic adenocarcinoma in a patient with a heavy history of familial digestive cancers.

## Patient and observation

It was a 66-year-old man admitted in our hospital in September 2012 with melena evolving since July 2012. The patient had heavy familial history of digestive cancers: his mother and one of his brothers died of colic cancer, his sister had colic polyposis, one of his maternal aunts had colic cancer and one other had gastric cancer. The patient himself was followed-up for an arterial hypertension and has undergone an adenomectomy in May 2010 for a benign hypertrophy of prostate. The patient has never undergone a screening for colorectal cancer. The patient didn't report epigastralgia, abdominal pain, diarrhea, constipation, vomiting, headache and loss of weight. The vital signs were stable. The abdominal examination didn't find any mass or hepatomegaly or splenomegaly or peritoneal collection. Rectal examination confirmed the melena. No superficial lymph node was found and the rest of the physical examination revealed no particularity. Initial blood tests showed hemoglobin of 10.9g/dL. Colonic endoscopy visualized a circumferential, ulcerated and obstructing formation at 20cm of the anal margin. The anatomopathological examination of the biopsy revealed a well differentiated adenocarcinoma.

**Figure 1:**
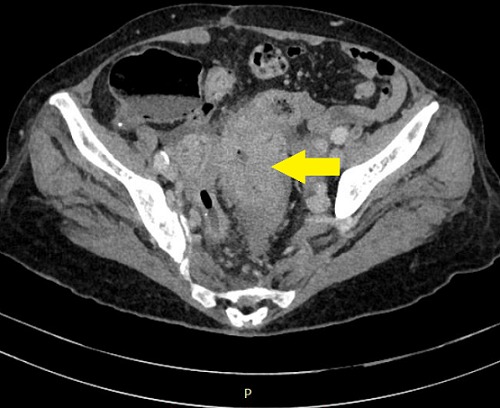
Abdominal computed tomography scan showing thickening of the sigmoid colon (indicated by arrow)

**Figure 2:**
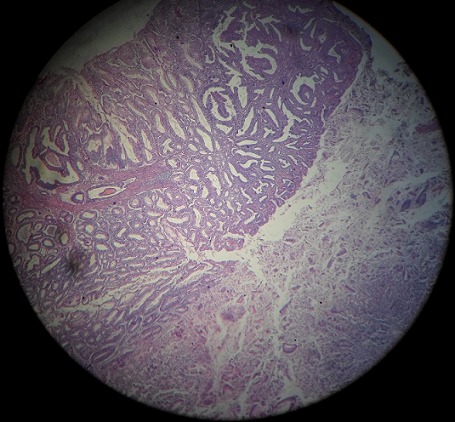
A photomicrograph of sigmoid adenocarcinoma colored by the hematoxylin-eosin magnified at 20 times

**Figure 3:**
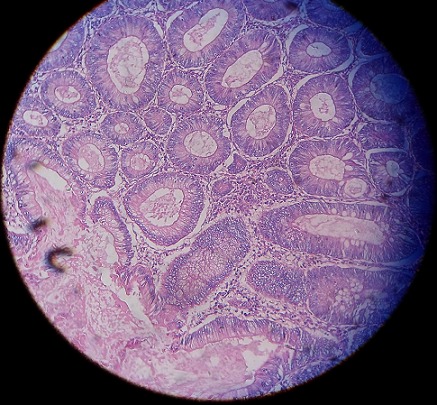
A photomicrograph of sigmoid adenocarcinoma colored by the hematoxylin-eosin magnified at 40 times

**Figure 4:**
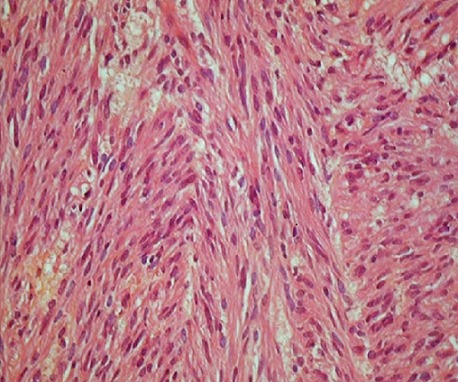
A photomicrograph of Jejunal stromal tumor colored by the hematoxylin-eosin magnified at 40 times

The abdominal computed tomography showed a circumferential formation of the sigmoid of 8cm height. There was no other mass or lymph node or peritoneal collection (Figure 1). The computed tomography of thorax had no particularities. Initial Carcino-Embryonar Antigen (CEA) was 10.3µg/L (normal value under 5µg/L) and initial Prostatic Specific Antigen (PSA) was 1.08ng/ml (normal). The patient underwent a left colectomy followed by immediate end-to-end anastomosis. A jejunal mass was discovered per-operatively and was suspected as a metastasis of the sigmoid adenocarcinoma. A jejuna segmentectomy was done and all operative pieces were sent to the pathologist. No immediate complication of the surgery was observed. The anatomopathological examination of the left colectomy confirmed the well-differentiated adenocarcinoma, measuring 8 x 5 x 2.5cm at 1cm of surgical margin classified as pT2N0M0. There were no positive nodes among 10 resected nodes (0N+/10N) (Figure 2, Figure 3). The examination of the jejunal fragment revealed a tumor measuring 1.9 x 1.5 x 1cm, with normal overlying mucosa, made of spindle cells, without nuclear atypia and without mitoses classified as low risk of recurrence (according to Miettinen criteria) (Figure 4). The stromal tumor was confirmed by the immunohistochemical study which showed positivity for CD117 and DOG1. The case was discussed in multidisciplinary reunion meeting and due to low risk of recurrence of the two tumors, no adjuvant therapy was needed. CEA control on October 2012 was 1.8µg/L (normal). The patient was followed-up until January 2018 and presented no signs of recurrence 64 months after the surgery of the both tumors.

## Discussion

The association of a GIST with another malignant tumor is estimated from 4.5% to 35% depending on series. Generally, the tumor associated with the GIST are gastrointestinal adenocarcinoma, prostatic adenocarcinoma, lymphoma, leukemia and breast cancer [5, 6]. Most cases of association of gastrointestinal adenocarcinoma and GIST are reported as solitary case report [3, 5, 7-9] and to our knowledge, only few series have been published [10, 11]. In reported cases, the age of patients presenting the association ranged from 47 to 84 years with a median age at the time of presentation of 68.6 years and both gender are concerned [7, 8, 10-12]. Here we report the first case of this association in a 66-year-old Malagasy male patient. Unlike our case which had a heavy history of gastrointestinal cancer, most reported cases of this association doesn't have familial history of cancer [3, 8, 13]. Though the association is generally sporadic cases, patients with familial history of colorectal cancer like ours should undergo screening for early detection of digestive tumors [14].

In most cases, like in ours, stromal tumors are an incidental finding per-operatively during surgery for the adenocarcinoma previously diagnosed by digestive endoscopy and are often suspected as small metastasis [10, 12, 15]. Few cases of GIST are discovered pre-operatively by computed tomography as part of the extension study of the adenocarcinoma [5, 10, 11]. Generally, the adenocarcinoma is discovered first, however few cases of adenocarcinoma secondarily discovered in patients followed for stromal tumors have been reported [8, 16]. Generally, the symptoms of the GIST are masked by the symptoms of the adenocarcinoma such as gastrointestinal bleeding, abdominal pain, vomiting and obstruction [3, 7, 16]. Clinical symptoms depend on the localization and the size of the tumors: tumors less than 2cm are generally asymptomatic while tumors with diameter more than 4cm are associated with symptomatic disease [17]. When associated with adenocarcinoma, the stromal tumor is generally small in size and has vague clinical symptoms such as poor appetite, bowel habit change or intermittent abdominal pain [3, 7, 18]. Thus, clinicians should be aware of the coexistence of malignancies and carry careful physical examination and imaging to improve their prognosis [3]. Per-operatively, careful examination and exploration of every lesion should be done and small lesions should not be considered as metastatic lesions [10].

The adenocarcinoma and the GIST can be localized in two different parts of the gastrointestinal tract or in the same part. The most frequently reported are association of a colic adenocarcinoma and a small bowel GIST like in our case [7, 19, 20], the association of a colic adenocarcinoma and a gastric GIST [3, 11, 15] and the association of a gastric adenocarcinoma and a gastric GIST [5, 10, 12, 15]. When localized in the same organ, they are generally well separated in different part of this organ. Exceptionally, Katsoulis *et al.* reported a collision tumor of an adenocarcinoma and a GIST in the stomach defined as the presence of adenocarcinoma cells within the GIST [12]. Many hypothesis were evoked about the pathogenesis of this association, as the presence of a causal relationship such as a same carcinogen that induce simultaneous proliferation of different cell lines but at the current state of science, further investigations are needed and their concurrence seems to be purely incidental [3, 7, 9, 11, 19]. The pathological examination of the surgical specimen of our patient showed a low risk adenocarcinoma associated with a low risk stromal tumor. When associated with adenocarcinoma, the stromal tumor is generally small in size ranging from 0.6 to 5cm, presents a low risk of invasion and recurrence compared to isolated GIST, with a mitotic index often less than 5 [5, 8, 10, 11]. This suggests a probable inhibition of the stromal tumor by the adenocarcinoma [18]. On the other side, many authors reported that in the presence of GIST, there was an apparent aggressiveness of adenocarcinoma which often presents with locally advanced stage, node invasions or/and metastatic spread [7, 11, 16]. Thus, clinicians should explore carefully any persistent digestive symptoms for the earliest detection of cancer.

The treatment and outcome depend on the localization and staging of the two associated tumors. For most reported cases, radical surgery was successful for the two associated tumors and like in our case, no adjuvant treatment was needed [3, 5, 8, 15]. For few cases with moderate to high risk of recurrence of GIST, imatinib was given as adjuvant treatment and Uracil-based chemotherapy was given as adjuvant treatment for advanced adenocarcinoma [7, 9, 16]. Our case distinguishes itself by the long follow-up of our patient which presented no sign of recurrence within 64 months after the radical surgery of the two tumors. Few authors reported no recurrence of both tumors during their follow-up ranging from 4 to 24 months after the radical resection [9, 13]. For most reported cases, the follow-up duration is not mentioned nor the issue of the patient [5, 7, 10, 11, 16]. Thus, the correct diagnosis of the tumors and its staging should be done carefully because it is the gage of an adapted treatment and a better prognosis.

## Conclusion

The association of a gastrointestinal adenocarcinoma and a stromal tumor is relatively uncommon. This association is often discovered incidentally during surgery for the adenocarcinoma due to the fact that his clinical and radiological signs mask the undergoing stromal tumor. Radical surgery of both tumors is the mainstay of therapy for their best control. Clinicians should be aware of the coexistence of malignancies and carry careful physical examination, imaging and per-operative examination to make patients have better prognosis. Follow-up should be done as much as possible to evaluate the outcome of the patients after the initial treatment.

## Competing interests

The authors declare no competing interest.

## References

[1] Miettinen M, Sarlomo-Rikala M, Lasota J (1999). Gastrointesinal stromal tumors: recent advances in understanding of their biology. Hum Pathol.

[2] Fletcher C, Berman J, Corless C, Gorstein F, Lasota J, Longley B Diagnosis of gastrointestinal stromal tumors: a consensus approach. J Hum Pathol.

[3] Chen C, Castellanos M, Ruch M, Hsu Y (2017). Gastrointestinal stromal tumor with synchronous colorectal adenocarcinoma. QJM Int J Med.

[4] Miettinen M, Lasota J (2003). Gastrointestinal stromal tumors (GISTs): definition, occurrence, pathology, differential diagnosis and molecular genetics. Pol J Pathol.

[5] Anis H, Selma K, Amine S, Houcine M, Montassar K (2017). Gastric synchronous stromal tumor and adenocarcinoma: A fortuitous association?. J Clin Exp Pathol.

[6] Yan Y, Li Z, Liu Y, Zhang L, Li J (2013). Coexistence of gastrointestinal stromal tumors and gastric adenocarcinomas. Tumor Biol.

[7] Chiu H, Huang T, Liu Y, Ko T, Lu N (2009). Synchronous ileal stromal tumor (GIST) and colonic adenocarcinoma. J Intern Med Taiwan.

[8] Dimitroulopoulos D, Foropoulou A, Xinopoulos D, Arnogiannaki N, Korkolis D, Tsamakidis K (2009). Synchronous occurrence of colorectal adenocarcinoma and colonic gastrointestinal stromal tumor (GIST). A case report. Ann Gastroenterol.

[9] Khoshnevis J, Rakhshan A, Sobhiyeh M, Gholizadeh B, Rahbari A, Adhami F (2013). Simultaneous gastric adenocarcinoma and gastrointestinal stromal tumor of the stomach: a case report. Iran J Cancer Prev.

[10] Cai R, Ren G, Wang D (2013). Synchronous adenocarcinoma and gastrointestinal stromal tumors in the stomach. World J Gastroenterol.

[11] Kaur R, Bhalla S, Nundy S, Jain S (2013). Synchronous gastric gastrointestinal stromal tumor (GIST) and other primary neoplasms of gastrointestinal tract: report of two cases. Ann Gastroenterol.

[12] Katsoulis I, Bossi M, Richman P, Livingstone J (2007). Collision of adenocarcinoma and gastrointestinal stromal tumor (GIST) in the stomach: report of a case. Int Semin Surg Oncol.

[13] Vasilakaki T, Koulia K, Tsavari A, Arkoumani E, Kouroumpas E, Pavlis A (2014). Synchronous gastric gastrointestinal stromal tumor and colon adenocarcinoma: A case report. Case Rep Oncol Med.

[14] Ransohoff D, Sandler R (2002). Screening for colorectal cancer. N Engl J Med.

[15] Lee J, Chung W, Lee K, Paik C, Kim H, Jun K (2010). Synchronous incidental occurence of gastric gastrointestinal stromal tumor and colon adenocarcinoma. Korean J Gastrointest Endosc.

[16] Efstathios P, Athanasios P, Papaconstantinou I, Alexandros P, Frangisca S, Sotirios G (2007). Coexistenec of gastrointestinal stromal tumor (GIST) and colorectal adenocarcinoma. World J Surg Oncol.

[17] Ludwig D, Traverso L (1997). Gut stromal tumors and their clinical behavior. Am J Surg.

[18] Liszka Ł, Zielinska-Pajak E, Pajak J, Golka D, Huszno J (2007). Coexistence of gastrointestinal stromal tumors with other neoplasms. J Gastroenterol.

[19] Gavriilidis P, Nikolaidou A (2015). Colon adenocarcinoma associated with synchronous extramural gastrointestinal stromal tumor (GIST) of the ileum. Am J Case Rep.

[20] Reda E, Ahmed J, Abderrahim E, Kawtar Z, Fouad Z, Zakia B (2015). Acute intestinal obstruction revealing synchronous gastrointestinal stromal tumors in a small bowel diverticulum and mucinous adenocarcinoma of the colon: a case report. Pan Afr Med J.

